# Pulmonary Nodules in Juvenile Systemic Sclerosis: A Case-Series from the National Registry for Childhood Onset Scleroderma (NRCOS)

**DOI:** 10.3390/diagnostics13122103

**Published:** 2023-06-17

**Authors:** Jonathan C. Li, Sameh Tadros, Franziska Rosser, Kathryn S. Torok

**Affiliations:** 1 UPMC Internal Medicine and Pediatrics Residency Program, University of Pittsburgh, Pittsburgh, PA 15224, USA; 2 Department of Pediatrics, Division of Radiology, School of Medicine, University of Pittsburgh, Pittsburgh, PA 15224, USA; 3 Department of Pediatrics, Division of Pulmonary Medicine, School of Medicine, University of Pittsburgh, Pittsburgh, PA 15224, USA; 4 Department of Pediatrics, Division of Rheumatology, School of Medicine, University of Pittsburgh, Pittsburgh, PA 15224, USA

**Keywords:** juvenile systemic sclerosis, pulmonary nodules, childhood lung disease, esophageal abnormalities, high-resolution computed tomography scan (HRCT), interstitial lung disease (ILD)

## Abstract

Background: Juvenile systemic sclerosis (jSSc) is a systemic inflammatory and fibrotic autoimmune disease. Adult guidelines recommend obtaining a screening high-resolution computed tomography scan (CT) at diagnosis. As these recommendations are adopted as standard of care for jSSc, increased screening with CT may lead to increased detection of nodules. The implications of nodules identified in jSSc are unclear and unreported. Methods: A retrospective chart review was performed on the prospectively enrolled National Registry for Childhood-Onset Scleroderma (NRCOS) cohort over an enrollment period of 20 years. Clinical associations with presence of nodules and nodule characteristics were investigated. Results: In this jSSc cohort, the prevalence of pulmonary nodules was 31% (*n* = 17 of 54). Nodule characteristics were heterogeneous, and most displayed stability over time. More participants with nodules had structural esophageal abnormalities, restriction, and reduced diffusing capacity on lung function tests, and follow-up imaging. Most participants had multiple nodules, and although most nodules were <5 mm, most participants had at least one nodule >5 mm. Conclusions: Pulmonary nodules are seen in children with jSSc and may be related to more severe disease and/or esophageal dysfunction. More work is needed to provide guidance on radiologic follow-up and clinical management of pulmonary nodules in jSSc.

## 1. Introduction

Juvenile systemic sclerosis (jSSc) is a systemic inflammatory and fibrotic autoimmune disease that frequently causes progressive interstitial lung disease (ILD). Pediatric SSc cohort studies have demonstrated ILD prevalence of 30–50%, similar to adults [1,2,3,4]. Given the high prevalence of ILD, availability of pharmacotherapeutics, and poor sensitivity of pulmonary function tests for making a diagnosis [5], adult guidelines recommend obtaining a screening high-resolution computed tomography scan (HRCT) of the chest at the time of SSc diagnosis [6].

While the goal of screening is to detect disease at an early stage, prior to significant fibrosis and at a stage where immunosuppression can be initiated, increased use of HRCT may result in more common detection of unclear pulmonary findings. At our tertiary care center, with a multidisciplinary Pediatric Scleroderma Clinic receiving nationwide referrals, we have appreciated several patients with reports of pulmonary nodules on HRCT. As pulmonary nodules are an infrequently categorized feature of either adult-onset SSc (aSSc) or jSSc populations, we sought to evaluate the prevalence and characteristics, both descriptive and clinical, of pulmonary nodules within jSSc patients enrolled in the National Registry for Childhood Onset Scleroderma (NRCOS).

## 2. Materials and Methods

### 2.1. Study Cohort

We performed a chart review of the prospective National Registry for Childhood-Onset Scleroderma (NRCOS) cohort. Registry details have been previously published [7,8]; in brief, NRCOS is a national registry which enrolls pediatric onset systemic sclerosis and localized scleroderma patients who are evaluated at the University of Pittsburgh Medical Center Children’s Hospital of Pittsburgh (UPMC-CHP) multidisciplinary Pediatric Scleroderma Center. UPMC-CHP is a single, tertiary center and receives nationwide referrals. All jSSc NRCOS participants, from inception 23 October 2002 to 31 December 2022, who had received chest computed tomography (CT) and a referring diagnosis of jSSc, were identified and medical records were reviewed.

### 2.2. Nodules

Chest CT scan reports were reviewed for any mention of the word nodule(s) or nodularity. Given the variation in available data, every effort was made to review the original radiology report for a specific study. In cases where the report was available, the entire report was evaluated. In some instances, only the final impression was available, which was also reviewed for the presence of the word nodule(s) or nodularity. Participants without mention of nodules were categorized in the no nodule group. Participants with mention of nodule(s) or nodularity were further reviewed by a single UPMC-CHP pediatric radiologist with special-expertise in chest imaging (S.T.). The majority of imaging was available within our EMR, as such imaging is typically uploaded during the referral/evaluation process. The pediatric radiologist (S.T.) reviewed available imaging to confirm the presence of nodule(s), and when present, characterized the nodules by zone (upper = apex to carina; middle = carina to lower inferior pulmonary veins; lower = lower inferior pulmonary veins to costophrenic sulcus), lung lobe (RUL = right upper lobe; RML = right middle lobe; RLL = right lower lobe; LUL = left upper lobe; LLL = left lower lobe), subatomic location (centrilobular, parenchymal, para-mediastinal, peri-fissure, peripheral, and subpleural), characteristics (tree-in-bud, calcified), and change across subsequent imagining (when available). A pediatric pulmonologist (F.R.) determined additional characteristics based on Hounsfield Unit (HU) (solid = HU > 0, ground glass opacity [GGO] = HU < 0, or part-solid = having both GGO and solid component) using radiologist-identified nodules.

### 2.3. Demographic & Clinical Information

Participants included satisfied the 2013 American College of Rheumatology and European League Against Rheumatism classification criteria (ACR/EULAR) for SSc and had at least 1 chest CT available in medical records [9].

To better assess the association of nodules and clinical characteristics of interest, we included both detailed demographic and clinical variables collected in the NRCOS database (K.T.) and supplemented this with a detailed chart review (J.L., F.R.) for select characteristics to include information about disease type and onset, serological antibody information, pulmonary disease, gastrointestinal (GI) disease, soft tissue and vascular disease, and usage of disease-modifying anti-rheumatic drug(s) (DMARDs).

We collected the following information for disease type and onset: age (in years) at onset of jSSc symptoms, age (in years) at initial CT chest or CT chest with nodule diagnosis, time from onset of jSSc symptoms to nodule diagnosis, or diagnosis of jSSc or jSSc-Overlap. We reviewed the reason for chest CT (e.g., screening, abnormal pulmonary function test or imaging, symptoms, unknown) and for any infectious disease management related to pulmonary nodules or at the time of CT scan acquisition, such as serologic testing (e.g., fungal markers), tuberculosis testing, bronchoscopy, and/or empirical antimicrobial therapy.

For serological typing, we reviewed ANA and extractable nuclear antigen antibodies (anti-Scl-70, anti-PM-Scl, anti-centromere, anti-RNA Pol III, anti-U1/U3RNP, and anti-histone) positivity.

For pulmonary disease, the presence of pulmonary symptoms (e.g., cough, dyspnea on exertion, shortness of breath at rest) at the time of initial chest CT, presence of interstitial lung disease and pulmonary function test (PFT) patterns from outside reports or as noted in referring provider evaluations were extracted. Pulmonary function tests were not reviewed for quality of technique and were infrequently available from outside centers. Definition of restriction or reduced diffusing capacity of carbon monoxide (DLCO) was not standardized and based upon outside interpretation.

For GI disease, we extracted data for esophageal dilatation, gastroesophageal reflux, and dysmotility at/nearest time of initial chest CT no greater than 1 year apart. We defined structural esophageal changes as any patulousness or dilation documented on any imaging (e.g., CT), functional testing (e.g., modified barium swallow, esophagram), and/or invasive testing (e.g., upper endoscopy). Presence of gastroesophageal reflux disease (GERD) was defined as having symptoms (e.g., heartburn), or evidence of reflux if demonstrated on pH probe/impedance testing, esophagram, and/or endoscopy findings. Gastrointestinal dysmotility was defined as symptoms of dysphagia, achalasia, early/prolonged satiety, abnormal peristalsis on endoscopy, abnormal esophageal peristalsis or pressures on manometry testing, and/or delayed gastric emptying (GE) demonstrated on GE study.

For vascular and soft tissue disease, we extracted information about the presence of Raynaud’s phenomena, nailfold capillary changes, digital pitting and/or ulceration, sclerodactyly and/or skin tightening, clubbing, myositis, and modified Rodnan skin score (mRSS) at or near the time of chest CT.

Use of DMARDs at the time of chest CT was collected. Drugs included hydroxychloroquine, mycophenolate mofetil, tacrolimus, cyclophosphamide, corticosteroids, IVIG, rituximab, and tocilizumab.

### 2.4. Statistical Analyses

Descriptive statistics were obtained for demographic and clinical variables. Bivariate analysis was performed using X^2^ or Fisher’s exact test, or Wilcoxon rank sum. Two-sided *p* values < 0.05 were considered significant. All analyses were performed using IBM SPSS Statistics v28.0.1.0 (142).

## 3. Results

### 3.1. Cohort

**Figure 1:**
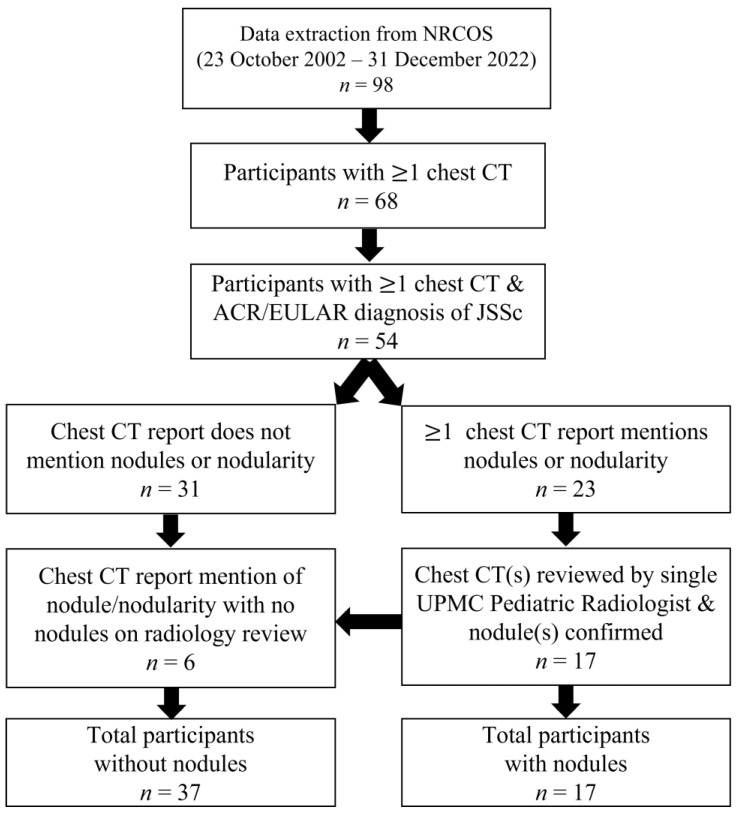
Flow diagram of patient inclusion and exclusion.

We identified 98 participants in the NRCOS registry that met criteria for jSSc or early jSSc diagnosis as of December 2022. Of these, 68 participants had at least one chest CT noted in our records, and of those, 54 fulfilled the 2013 ACR/EULAR SSc criteria. Those excluded either met partial criteria for SSc or were later diagnosed as having mixed or undifferentiated connective tissue disease. Of those defined as jSSc, 31 (57%) did not have nodule(s) or nodularity identified on chest CT report. Twenty-three participants had reports of nodule(s) or nodularity on at least one chest CT report; however, 17 (31%) had the presence of nodules confirmed on radiographic review. The six participants with reports of nodule(s)/nodularity but absence of nodules on imaging review were re-classified as absence of nodules (Figure 1).

### 3.2. Clinical Characteristics

We compared selected characteristics between JSSc participants with (*n* = 17) and without nodules (*n* = 37). The group (*n* = 54) mean age of JSSc-onset and initial CT chest was 10.1 (Standard Deviation [SD] 3.8) and 12.6 (SD 4.1) years, respectively, and did not differ between groups (Table 1).

Participants with nodules had reduced time from onset of symptoms to initial chest CT and a higher prevalence of jSSc-overlap; however, neither was significant. Participants with nodules had a higher percentage of follow-up CT imaging (88%) compared to children without nodules (38%) (*p* < 0.001). Infectious disease evaluation was only initiated for children with nodules (*n* = 3), and such evaluations were low even among those with nodules (18%).

Most participants demonstrated ANA positivity (89%), which did not differ between children with and without nodules. We found no jSSc-specific-antibody differences between children with and without nodules.

Participants with nodules had a higher prevalence of interstitial lung disease (41%) compared to children without (27%), although this was not significant. A greater percentage of children with nodules had abnormal pulmonary function test (restrictive pattern and reduced DLCO), *p* < 0.01 for both). Overall, few children had documented cough or dyspnea on exertion; however, three children, all in the nodule group, had documented chest pain.

Structural esophageal abnormalities were more prevalent in children with nodules (41% vs. 5%, *p* = 0.001). Gastroesophageal reflux and upper GI dysmotility were more frequent in children with nodules compared to those without, but neither was statistically significantly different between groups. Interestingly, PPI or H2 blocker usage did not differ between groups despite increased structural abnormalities in children with nodules.

Nearly all participants had Raynaud’s (96%), nailfold capillary changes (89%), and sclerodactyly (78%), which did not differ between groups. The presence of digital ulceration and/or fingertip pitting was less frequent in children with nodules (47% vs. 78% in children without nodules, *p* = 0.02). More children with nodules had evidence of myositis (64% vs. 35%), but this was not significant (*p* = 0.08). Average mRSS was overall low for the cohort (mean 9.13, SD 11.0), but similar to that reported in a recent international jSSc cohort (*n* = 150) [1] and did not differ between groups.

Fewer children with nodules were receiving at least one DMARD compared to children without nodules (52% vs. 73%, *p* = 0.15); however, this was not statistically significantly different. We found no difference with specific DMARDs between groups.

### 3.3. Nodule Characteristics

Nodule characteristics by participant (*n* = 17) are shown in Table 2. Over half (53%) of participants had nodules discovered on chest CT obtained for screening and close to one-third had nodules discovered on chest CT obtained for abnormal pulmonary function tests. Forty percent (*n* = 7) had documented pulmonary symptoms at the time of chest CT acquisition, with two participants reporting symptoms consistent with acute infection (Participants 1 and 8). Most participants had repeat CT chest imaging (*n* = 14, 82%), with an average of 3.4 CT scans per participant. Interval monitoring ranged from 0.6 to 4.5 years.

Only one participant had a solitary nodule (Participant 9), with most participants having multiple nodules (*n* = 16, 94%). Most nodules were less than or equal to 5 mm, however most participants (*n* = 10, 59%) had at least one nodule >5 mm in size (Table 3). Three participants (8, 13, 14) had tree-in-bud nodular opacities, which resolved completely in the two participants with follow-up imaging (13 and 14) and was associated with infectious symptoms in one participant (8). Most participants had at least one GGO nodule (*n* = 15, 88%) and half had at least one solid nodule (*n* = 9, 53%). Part-solid nodules were identified in four participants (24%).

There was generally an equal distribution of lung zone involvement (upper, middle, lower). Five participants (29%) had at least one nodule in all three zones. The majority of participants had at least one nodule in the lower lobes (71%), with the left lower lobe being the most common (*n* = 12, 71%) followed by the right lower lobe (*n* = 11, 65%), with bilateral lower lobe involvement in eight participants (47%). Within the lung, most participants had at least one nodule located in located subpleurally (*n* = 14, 82%) and peripherally (*n* = 11, 65%). Less frequently observed nodule locations included parencyhmal (*n* = 5, 29%) and centrilobular (*n* = 2, 12%).

Of participants with repeat imaging (*n* = 14), most nodules remained stable (*n* = 13, 93%). No nodules were noted to increase in size, 4 participants were noted to develop new nodules (Participants 4, 5, 7, and 11), and 13/14 participants demonstrated stability to mild decrease in size or resolution of nodules. The only participant to have complete resolution of all nodules had tree-in-bud nodularity (Participant 14). Of the four participants who developed new nodules, all had progressive gastrointestinal disease. No nodules were biopsied, and no participants were diagnosed with malignancy. A summary of nodule characteristics is provided in Table 3.

## 4. Discussion

Pulmonary nodules are a feature of juvenile systemic sclerosis, with a prevalence of 31% (*n* = 17 of 54) in our cohort. Nodules were heterogeneous in size, location, and appearance. Most participants had at least one nodule that was GGO, subpleural, and present in a lower lobe. Most nodules displayed stability over time, and most participants had more than one nodule. Time between symptom onset and chest CT was lower for children with nodules, suggesting possible more severe or extensive disease at diagnosis. Children with nodules had a higher prevalence of abnormal pulmonary function tests and structural esophageal abnormalities, which may also signify more severe or extensive disease. A higher percentage of participants with nodules had ILD and jSSc-overlap, although this was not significant and may be limited by our small sample size.

To our knowledge, this is the first dedicated description of pulmonary nodules in jSSc, thus limiting comparison to prior studies. The only mention of pulmonary nodules in jSSc we identified was in a study by Ambartsumyan et al., who identified an association between decreased PFTs in jSSc and esophageal abnormalities. They reported a nodule prevalence of 19% (*n* = 3/16) on chest CT and did not find any associations between nodules and esophageal abnormalities [10]. In contrast, we observed that esophageal abnormalities were more common among participants with nodules.

The true prevalence of pulmonary nodules in children is unknown; however, studies in healthy children have reported ranges between 1.2 and 75% [11]. We are therefore unable to determine if a prevalence of 31% in our jSSc cohort is more or less common compared to incidental nodules in children. Such information would be helpful in ascertaining if nodules are within the range expected for children, regardless of jSSc. Additionally, we do not have a non-jSSc pediatric rheumatologic disease cohort with routine CT chests for comparison.

Pulmonary nodules have been described in adult-onset SSc, although they are not a commonly described pulmonary feature [12]. Limited reports on aSSc pulmonary nodules suggest a high prevalence, between 70 and 87% among patients with an abnormal CT, but these studies are limited by small sample sizes [13,14]. Remy-Jardin et al. provided an extensive report describing nodules in aSSc [14]. They classified nodules into two subcategories: parenchymal micronodules and subpleural micronodules (both <7 mm in width). They reported that among aSSc patients with an abnormal pulmonary CT without honeycombing, prevalence of parenchymal nodules was 46% and subpleural nodules was 85%. If honeycombing was present, there was a higher prevalence of parenchymal nodules at 74%, and a similar prevalence of subpleural nodules at 89%. They observed that subpleural nodules were predominantly present bilaterally, in upper lobes, and posterior regions. No nodules were calcified. Two of eight patients had resolution of micronodules.

Findings from our current study are similar, showing that most participants had at least one subpleural nodule. Remy-Jardin et al. reported that six of eight patients with parenchymal micronodules had new-onset or progression of honeycombing in that same area, suggesting they are related to the same process that produces ILD. They hypothesized that parenchymal micronodules reflected lymphoid hyperplasia and that subpleural micronodules reflected lymphoid aggregates at the pleural–parenchymal interphase [14]. Unlike their study, no nodules evolved into regions of honeycombing in our population, and most nodules remained stable over time. The study by Remy-Jardin et al. was conducted over three decades ago, and our knowledge of DMARDs for SSc treatment has since considerably evolved. It is therefore unknown whether the routine usage of DMARDs within our population prevented development of ILD.

A restrictive pattern on PFT and reduced DLCO were more common in participants with nodules compared to those without. This may be disproportionate, as, although ILD was more common in participants with nodules versus those without, it did not reach statistical significance. The reason for abnormal PFT findings is unclear but could be related to overall more severe disease, increased prevalence of overlap with myositis (which can impact respiratory muscles), or as Remy-Jardin et al. speculated, nodules could represent pulmonary jSSc involvement. The presence of the nodules themselves would not be sufficient to cause either restriction or reduced DLCO.

As none of our participants underwent nodule biopsy, we are unable to determine the underlying pathophysiology of the nodules. The presence of tree-in-bud is a nonspecific finding that is associated with infection and inflammation. Given that the tree-in-bud nodules resolved in the two participants with follow-up imaging, we suspect they are different from the nodules seen in the other 14 participants. While nodule appearance is non-specific, ground glass opacities are typically thought to reflect inflammation, infection, micro-atelectasis, or scarring [15]. Potential sources of inflammation in jSSc include retrograde aspiration from esophageal dysfunction, underlying inflammation due to jSSc itself, or infection due to immunosuppression. Without pathological clarity, we are unable to determine the etiology of the nodules; however, infection is less likely given the absence of infectious symptoms and persistence of nodules over time. We suspect some of the nodules in our series were related to infection, particularly for participant 8, whose tree-in-bud nodules were associated with fever and subsequently resolved, and for participant 1, who had upper respiratory symptoms at the time of nodule diagnosis but no subsequent imaging.

We observed a higher percentage of esophageal dysfunction in participants with nodules compared to those without. It is possible that nodules are related to more severe disease, as one would expect with more esophageal involvement. Alternatively, it is possible that esophageal dysfunction increases micro-aspiration, thereby causing pulmonary insults and nodules. Although no equivalent studies have been performed in pediatrics, adult studies have recognized the association between gastroesophageal disease and ILD in SSc and that the severity of gastroesophageal disease is underappreciated based on symptomatology [16,17,18,19].

Management of jSSc patients after identification of a nodule remains unclear. The Fleischner Society Guidelines are generally referred to for guidance on managing incidental pulmonary nodules in patients >35 years of age [20] and are not appropriate for children, in whom the risk for malignancy is low [15]. Accordingly, in 2015 the Society of Pediatric Radiology (SPR) Thoracic Imaging Committee published a general guideline for pulmonary nodules <3 cm in size [15]. The guidelines provided a broad framework for approaching incidental pulmonary nodules. Liang and Lee (2022) provided additional guidance by building off the SPR guideline based on interval literature and suggested that any nodule <5 mm does not require dedicated follow-up imaging if not warranted clinically [11]. An important distinction is the definition of incidental nodule, defined as a nodule in a healthy child without history of immune deficiency, malignancy, or congenital pulmonary airway malformation. However, neither the Fleischner Society Guidelines nor the SPR guidelines mention pulmonary nodules in persons with systemic autoimmune/rheumatologic conditions.

Pulmonary nodules are well-known features in a number of autoimmune/rheumatologic conditions including vasculitides (e.g., giant cell arteritis, Behcet’s disease, ANCA-associated, Kawasaki disease), Crohn’s disease, IgG4-related disease, systemic lupus erythematosus, and Evan’s syndrome [21,22,23,24,25,26,27]. Additionally, persons with aSSc are also at risk for pulmonary malignancy (adenocarcinoma) [28], although it is less clear whether jSSc is associated with similar risk. To our knowledge, there has been only one case report of jSSc associated with pulmonary adenocarcinoma, who also had a congenital pulmonary airway malformation which is known to be a risk for malignancy [29]. Thus, we are hesitant to refer to nodules as incidental in jSSc, as the true incidence, etiology, and significance remains unclear. However, our study does support that most nodules, in patients receiving specialist care, are stable over time.

The main limitation to our study is sample size. This is predominantly due to the rarity of jSSc and that most participants within our cohort are referred to our center for expert opinion, limiting our access to complete medical records for many of them. We also unexpectedly observed a significantly lower prevalence of digital ulceration/pitting in the nodule cohort. However, it should be noted that this was at time of CT and we suspect that liberalizing our criteria of digital pitting/ulceration to any time point would eliminate this finding. Digital ulceration and pitting are well associated with disease severity, reduced pulmonary function, and ILD in SSc [30,31,32]. Further study of vasculopathy may help discern this association, and although our cohort showed general capillary nailfold changes (NFC), they were not evaluated using the standard SSc approach which categorizes NFC into early, active, and late to allow the comparison with presences/absence of nodules. Some general caution is warranted with interpretation of PFT abnormalities as the studies were from varying testing facilities, represent varying prediction equations, were not reviewed for reproducibility and acceptability, and did not have a standardized definition for abnormality.

## 5. Conclusions

Pulmonary nodules are commonly found in a national registry of juvenile systemic sclerosis. These nodules were associated with structural esophageal abnormalities and abnormal pulmonary function tests. A higher percentage of participants with nodules also had ILD and jSSc-overlap, but this was not significant. Patients with jSSc and pulmonary nodules may require closer evaluation for aspiration. It is anticipated that nodules will be more frequently encountered given recommendations for HRCT of the chest at diagnosis, thus creating diagnostic and management dilemmas. Given the paucity of evidence, guidelines cannot be offered regarding follow-up of these nodules. Further studies are needed, although such will likely be hindered by rarity of jSSc and under-adoption of adult guidelines for screening HRCT. Low-dose chest CT scans may be an important tool in the future for both diagnosis and serial monitoring.

## Figures and Tables

**Table 1:** Select characteristics of juvenile systemic sclerosis registry participants with at least one chest CT scan (*n* = 54).

Clinical Characteristics	Nodule(s) (*n* = 17)	No Nodules (*n* = 37)	*p* Value
Age of JSSc-onset, years	9.7 (±4.0)	10.3 (±3.7)	0.80
Age at initial CT or nodule diagnosis, years *	11.6 (±3.6)	13.0 (±4.2)	0.36
Time from jSSc onset to nodule diagnosis, years	1.8 (±1.3)	2.7 (±3.5)	0.08
Diagnosed with jSSc-overlap disease	11 (64)	14 (38)	0.08
Infectious disease evaluation at time of initial nodule(s)	3 (18)	0 (0)	**0.01**
Received follow-up CT	15 (88)	14 (38)	**<0.001**
**Serologies**			
ANA positive	14 (82)	34 (92)	0.30
Anti-Scl-70 positive	5 (29)	13 (35)	0.68
Anti-PM-Scl positive	5 (29)	7 (19)	0.39
Anti-centromere positive	2 (12)	1 (3)	0.18
Anti-RNA Pol III positive	0 (0)	2 (5)	0.33
Anti-U1/U3RNP positive	3 (18)	6 (16)	0.90
Anti-histone positive	0 (0)	1 (3)	0.49
Antibody negative	3 (18)	10 (27)	0.45
**Pulmonary symptoms**			
None	10 (59)	28 (76)	0.21
Cough	3 (18)	3 (8)	0.30
Dyspnea on exertion	3 (18)	5 (14)	0.69
Shortness of breath, rest	0 (0)	2 (5)	0.33
PFT with restrictive pattern (*n* = 48)	9 (56)	5 (11)	**0.004**
PFT with reduced DLCO (*n* = 39)	7 (58)	6 (22)	**0.03**
Diagnosed with interstitial lung disease	7 (41)	10 (27)	0.30
**Gastrointestinal disease features**			
Structural esophageal abnormalities	7 (41)	2 (5)	**0.001**
GERD	12 (71)	20 (54)	0.25
Upper GI dysmotility	8 (47)	13 (35)	0.40
On PPI or H2B therapy	5 (29)	13 (35)	0.68
**Soft tissue and vascular disease features**			
Raynaud’s	16 (94)	36 (97)	0.57
Nailfold capillary abnormalities	15 (88)	33 (89)	0.92
Digital ulceration/pitting, yes	8 (47)	29 (78)	**0.02**
Sclerodactyly	15 (88)	27 (73)	0.21
Clubbing	1 (6)	1 (3)	0.57
Myositis	10 (59)	13 (35)	0.10
Modified Rodnan skin score (mRSS) (*n* = 46)	10.5 (±11.4)	8.5 (±11.0)	0.63
**DMARD at time of CT**			
On any DMARD at time of nodule diagnosis	9 (52)	27 (73)	0.15
Mycophenolate mofetil	4 (24)	14 (38)	0.30
Methotrexate	4 (24)	13 (35)	0.39
Prednisone	4 (24)	11 (30)	0.64
Cyclophosphamide	1 (6)	2 (5)	0.94
Hydroxychloroquine	5 (29)	10 (27)	0.86
Intravenous Immune Globulin	3 (18)	5 (14)	0.69
Rituximab	1 (6)	3 (8)	0.77

Results displayed as n (%) or mean (SD). *p* value obtained from X^2^ or Fisher’s exact test (where appropriate), or Wilcoxon rank sum. **Bold** = *p* value < 0.05. * Age of initial chest computed tomography (CT) for those without nodules and age of initial CT chest demonstrating nodules for those found to have nodules.

**Table 2:** Characteristics of nodules by juvenile systemic sclerosis patient.

Patient	Reason for CT	# CT Scans	Interval (Years)	# Nodules	Any >5 mm	Lung Zone	Lung Lobe	Location	Characteristic	Course Over Time	Pulmonary Symptoms at Initial CT	ILD
1 *SCL 70+*	Screening	1	-	Multiple (3)	No	U, M	RUL	Parenchyma, centrilobular	GGO	-	URI symptoms	No
2 *Overlap*, *negative antibodies*	Screening	2	0.6	Multiple	Yes	U, M	RUL, RLL, LUL	Centrilobular, subpleural	GGO	Stable, resolution RUL nodules	None	No
3 *Overlap*, *+PM SCL*	Screening	3	3.9	Multiple (3)	No	M, L	RML, LLL	Peripheral, subpleural	Solid	Stable, interval calcification of 1 nodule	Exertional dyspnea	No
4 *SCL 70+*	Abnormal PFT	5	4.2	Multiple (6)	Yes	M, L	RLL, LUL, LLL	Peripheral, subpleural	Solid, GGO	Stable, development of 2 new calcified nodules, interval calcification of 1 nodule	Cough, exertional dyspnea	Yes
5 *SCL 70+*, *RNP3+*	Screening	2	2.3	Multiple (5)	No	U, M, L	RUL, RML, LUL, LLL	Peripheral, peri-fissure, parenchymal	GGO	Stable, development of 1 new subpleural nodule	None	Yes
6 *Overlap*, *Oklahoma panel negative*	Abnormal PFT	3	2.4	Multiple (8)	No	U, M, L	RUL, RML, RLL, LUL	Peripheral, paramediastinal, peri-fissure, subpleural	GGO, solid, part-solid	Stable	None	No
7 *Negative Oklahoma panel*	Unknown	4	4.2	Multiple (7)	Yes	U, M, L	RUL, RLL, LLL	Parenchymal, peripheral, subpleural	GGO, solid, linear	Stable, large 9 mm solid nodule resolved, 2 new nodules developed with resolution of 1	Cough (‘slight’)	Yes
8, *Overlap/SLE*, *SCL 70+*	Abnormal chest xray	2	1.1	Multiple (tree-in-bud)	Yes	M, L	LLL	Peri-fissue, peripheral, subpleural	Tree-in-bud, part-solid, GGO	Tree-in-bud resolved, decreased extent of nodules, stable	Cough, fever, exertional dyspnea	Yes
9 *Overlap*, *PM/SCL 100+*	Abnormal PFT	3	1.9	Solitary	Yes	M	LLL	Peri-fissure	GGO	Stable, minimal decrease size	None	Yes
10 *Antibodies unknown*	Unknown	4	2.4	Multiple	Yes	L *	RML, RLL, LLL *	Peripheral, subpleural	GGO	Stable, minimal decrease size	Unknown	Yes
11 *Overlap*, *PM/SCL 100+*	Screening	3	2.1	Multiple (3)	Yes	U, L	RLL, LUL, LLL	Parenchymal, subpleural	Solid, part-solid (with halo)	Stable, 2 nodules (solid) on screening CT chest. Interval development of 6 mm nodule with halo which resolved	None	No
12 *Overlap*, *PM/SCL 100+*	Screening	1	-	Multiple (6)	No	U, L	RML, RLL, LLL	Parenchymal, peri-fissure, subpleural	GGO, solid	-	None	No
13 *SCL 70+*	Screening	1	-	Multiple (tree-in-bud)	Yes	U	RUL	Peri-fissure, peripheral, subpleural	GGO	-	None	No
14 *U3RNP +* *Overlap*	Screening	4	1.6	Multiple (tree-in-bud)	Yes	U, M	RUL, RLL	Peri-fissure, subpleural	GGO, part-solid, solid	Resolution of all nodules	None	No
15 *Overlap*, *PM/SCL 100+*	Abnormal PFT	5	2.1	Multiple (2)	No	M, L	RLL, LLL	Peripheral, subpleural	GGO, solid	Stable	Unknown	Yes
16 *Overlap*, *U3RNP +*, *anticentromere+*	Screening	2	0.8	Multiple (9)	Yes	U, M, L	RUL, RML, RLL, LUL, LLL	Peripheral, subpleural	GGO	Stable, 1 nodule decreased in size, 1 nodule resolved.	Fatigue, TB testing negative	No
17 *Overlap*, *U1 RNP*	Abnormal PFT	4	4.5	Multiple (4)	No	U, M, L	RUL, RML, RLL, LLL	Peripheral, subpleural	GGO, Solid	Stable	Intermittent pleuritic chest pain	Yes

Definitions: # nodules is categorized as solitary (1 nodule) vs. multiple (>1 nodule). Where available, the maximum number of nodules is presented; Any >5 mm is categorized “yes” if 1 or more nodules were >5 mm and “no” if all nodules were <5 mm. Lung zones are defined as U = upper (apex to carina), M = middle (carina to inferior pulmonary veins), L = lower (inferior pulmonary veins to costophrenic sulcus). Lung lobes: RUL: right upper lobe, RML: right middle lobe, RLL: right lower lobe, LUL: left upper lobe and/or lingula, LLL: left lower lobe. Location is defined as: parenchymal, peripheral, subpleural, centrilobular, peri-fissure. Characteristics are defined as solid (Hounsfield unit >0); ground glass opacities (GGO) defined as Hounsfield unit <0; part-solid defined as having both solid and GGO components; calcified; linear. Note that characteristics are provided if any nodule had the characteristic (for example, in a patient with 6 nodules, if 5 were GGO and 1 was solid, the characteristics would read GGO, solid). Presence of ILD is any signs consistent with interstitial lung disease on CT chest. * Two CT scans were available for review—a CTA and CT abdomen with lung bases. Patients 1, 12 and 13 only had one timepoint, hence the missing data items denoted as “-“. Definitions: # nodules is categorized as solitary (1 nodule) vs. multiple (>1 nodule). Where available, the maximum number of nodules is presented; Any >5 mm is categorized “yes” if 1 or more nodules were >5 mm and “no” if all nodules were <5 mm. Lung zones are defined as U = upper (apex to carina), M = middle (carina to inferior pulmonary veins), L = lower (inferior pulmonary veins to costophrenic sulcus). Lung lobes: RUL: right upper lobe, RML: right middle lobe, RLL: right lower lobe, LUL: left upper lobe and/or lingula, LLL: left lower lobe. Location is defined as: parenchymal, peripheral, subpleural, centrilobular, peri-fissure. Characteristics are defined as solid (Hounsfield unit >0); ground glass opacities (GGO) defined as Hounsfield unit <0; part-solid defined as having both solid and GGO components; calcified; linear. Note that characteristics are provided if any nodule had the characteristic (for example, in a patient with 6 nodules, if 5 were GGO and 1 was solid, the characteristics would read GGO, solid). Presence of ILD is any signs consistent with interstitial lung disease on CT chest. * Two CT scans were available for review—a CTA and CT abdomen with lung bases. Patients 1, 12 and 13 only had one timepoint, hence the missing data items denoted as “-”.

**Table 3:** Prevalence of nodule characteristics.

Nodule Characteristic	*n* (Total = 17)	Percent
Size		
>5 mm	10	59
Pattern		
Ground glass	15	88
Solid	9	53
Semi-solid	4	24
Tree-in-bud	3	18
Lung Zone		
Upper	11	65
Middle	13	77
Lower	12	71
All zones	5	29
Lobe		
RUL	9	53
RML	7	41
RLL	11	65
LUL	6	35
LLL	12	71
Right side only	3	18
Left side only	2	12
Bilateral	12	71
Bilateral LL	8	47
Location		
Subpleural	14	82
Peripheral	11	65
Peri-fissure	7	41
Parenchymal	5	29
Centrilobular	2	12

Characteristics refer to at least one nodule per participant and do not reflect individual nodules. For example, eleven participants had at least one nodule in the upper lung zone.

## Data Availability

Data are contained within the article.

## References

[1] Foeldvari I., Klotsche J., Kasapcopur O., Adrovic A., Terreri M.T., Sakamoto A.P., Stanevicha V., Sztajnbok F., Anton J., Feldman B. (2022). Differences Sustained Between Diffuse and Limited Forms of Juvenile Systemic Sclerosis in an Expanded International Cohort. Arthritis Care Res..

[2] Foeldvari I., Tyndall A., Zulian F., Muller-Ladner U., Czirjak L., Denton C., Kowal-Bielecka O., Bancel D.F., Matucci-Cerinic M. (2012). Juvenile and Young Adult-Onset Systemic Sclerosis Share the Same Organ Involvement in Adulthood: Data from the EUSTAR Database. Rheumatology.

[3] Scalapino K., Arkachaisri T., Lucas M., Fertig N., Helfrich D.J., Londino A.V., Steen V.D., Medsger T.A. (2006). Childhood Onset Systemic Sclerosis: Classification, Clinical and Serologic Features, and Survival in Comparison with Adult Onset Disease. J. Rheumatol..

[4] Stevens B.E., Torok K.S., Li S.C., Hershey N., Curran M., Higgins G.C., Moore K.F., Egla Rabinovich C., Dodson S., Stevens A.M. (2018). Clinical Characteristics and Factors Associated with Disability and Impaired Quality of Life in Children with Juvenile Systemic Sclerosis: Results from the Childhood Arthritis and Rheumatology Research Alliance Legacy Registry. Arthritis Care Res..

[5] Foeldvari I., Klotsche J., Hinrichs B., Helmus N., Kasapcopur O., Adrovic A., Sztajnbok F., Terreri M.T., Anton J., Smith V. (2022). Underdetection of Interstitial Lung Disease in Juvenile Systemic Sclerosis. Arthritis Care Res..

[6] Rahaghi F.F., Hsu V.M., Kaner R.J., Mayes M.D., Rosas I.O., Saggar R., Steen V.D., Strek M.E., Bernstein E.J., Bhatt N. (2023). Expert Consensus on the Management of Systemic Sclerosis-Associated Interstitial Lung Disease. Respir. Res..

[7] Prasad S., Zhu J.L., Schollaert-Fitch K., Torok K.S., Jacobe H.T. (2021). An Evaluation of the Performance of Current Morphea Subtype Classifications. JAMA Derm..

[8] Ardalan K., Zigler C.K., Torok K.S. (2017). Predictors of Longitudinal Quality of Life in Juvenile Localized Scleroderma. Arthritis Care Res..

[9] van den Hoogen F., Khanna D., Fransen J., Johnson S.R., Baron M., Tyndall A., Matucci-Cerinic M., Naden R.P., Medsger T.A., Carreira P.E. (2013). 2013 Classification Criteria for Systemic Sclerosis: An American College of Rheumatology/European League against Rheumatism Collaborative Initiative. Arthritis Rheum..

[10] Ambartsumyan L., Zheng H.B., Iyer R.S., Soares J., Henstorf G., Stevens A.M. (2019). Relationship Between Esophageal Abnormalities on Fluoroscopic Esophagram and Pulmonary Function Testing in Juvenile Systemic Sclerosis. Arthritis Care Res..

[11] Liang T.I., Lee E.Y. (2022). Pediatric Pulmonary Nodules: Imaging Guidelines and Recommendations. Radiol. Clin..

[12] Ahuja J., Arora D., Kanne J.P., Henry T.S., Godwin J.D. (2016). Imaging of Pulmonary Manifestations of Connective Tissue Diseases. Radiol. Clin. North Am..

[13] Kim E.A., Johkoh T., Lee K.S., Ichikado K., Koh E.-M., Kim T.S., Kim E.Y. (2001). Interstitial Pneumonia in Progressive Systemic Sclerosis: Serial High-Resolution CT Findings with Functional Correlation. J. Comput. Assist. Tomogr..

[14] Remy-Jardin M., Remy J., Wallaert B., Bataille D., Hatron P.Y. (1993). Pulmonary Involvement in Progressive Systemic Sclerosis: Sequential Evaluation with CT, Pulmonary Function Tests, and Bronchoalveolar Lavage. Radiology.

[15] Westra S.J., Thacker P.G., Podberesky D.J., Lee E.Y., Iyer R.S., Hegde S.V., Guillerman R.P., Mahani M.G. (2015). The Incidental Pulmonary Nodule in a Child. Part 2: Commentary and Suggestions for Clinical Management, Risk Communication and Prevention. Pediatr. Radiol..

[16] Volkmann E.R., Tashkin D.P., Leng M., Kim G.H.J., Goldin J., Roth M.D. (2022). Association of Symptoms of Gastroesophageal Reflux, Esophageal Dilation, and Progression of Systemic Sclerosis-Related Interstitial Lung Disease. Arthritis Care Res..

[17] Savarino E., Bazzica M., Zentilin P., Pohl D., Parodi A., Cittadini G., Negrini S., Indiveri F., Tutuian R., Savarino V. (2009). Gastroesophageal Reflux and Pulmonary Fibrosis in Scleroderma: A Study Using PH-Impedance Monitoring. Am. J. Respir. Crit. Care Med..

[18] Raja J., Ng C.T., Sujau I., Chin K.F., Sockalingam S. (2016). High-Resolution Oesophageal Manometry and 24-Hour Impedance-PH Study in Systemic Sclerosis Patients: Association with Clinical Features, Symptoms and Severity. Clin. Exp. Rheumatol..

[19] Murdaca G., Caprioli S., Tonacci A., Billeci L., Greco M., Negrini S., Cittadini G., Zentilin P., Ventura Spagnolo E., Gangemi S. (2021). A Machine Learning Application to Predict Early Lung Involvement in Scleroderma: A Feasibility Evaluation. Diagnostics.

[20] Bueno J., Landeras L., Chung J.H. (2018). Updated Fleischner Society Guidelines for Managing Incidental Pulmonary Nodules: Common Questions and Challenging Scenarios. RadioGraphics.

[21] Higuchi Y., Ochi M., Shimizu J., Furujo M. (2020). A 9-Year-Old Girl with Kawasaki Disease and Pulmonary Nodules. Clin. Rheumatol..

[22] Namireddy M.K., Consul N., Sher A.C. (2021). FDG-Avid Pulmonary Nodules and Tracheobronchial Mural Inflammation in IgG4-Related Disease. Clin. Nucl. Med..

[23] Cuceoglu M.K., Ozen S. (2021). Pulmonary Manifestations of Systemic Vasculitis in Children. Pediatr. Clin. North Am..

[24] Freeman A.F., Crawford S.E., Finn L.S., López-Andreu J.A., Ferrando-Monleón S., Pérez-Tamarit D., Cornwall M.L., Shulman S.T., Rowley A.H. (2003). Inflammatory Pulmonary Nodules in Kawasaki Disease. Pediatr. Pulmonol..

[25] Pincez T., Neven B., Le Pointe H.D., Varlet P., Fernandes H., Gareton A., Leverger G., Leblanc T., Chambost H., Michel G. (2019). Neurological Involvement in Childhood Evans Syndrome. J. Clin. Immunol..

[26] Cao W., Deng X., Xu C., Wang X., Yu Y., Xu X., Li J., Xiao Y. (2021). Crohn’s Disease with Pulmonary Granuloma in a Child: A Case Report and Review of the Literature. Transl. Pediatr..

[27] Tamai H., Nishina N., Takeuchi T. (2019). Pulmonary Vein Vasculitis Presenting as Multiple Pulmonary Nodules in a Patient with Systemic Lupus Erythematosus. J. Rheumatol..

[28] Lepri G., Catalano M., Bellando-Randone S., Pillozzi S., Giommoni E., Giorgione R., Botteri C., Matucci-Cerinic M., Antonuzzo L., Guiducci S. (2022). Systemic Sclerosis Association with Malignancy. Clin. Rev. Allergy Immunol..

[29] Aliyeva A., Adrovic A., Ocak S., Batur Ş., Yıldız M., Haşlak F., Köker O., Şahin S., Barut K., Kasapçopur Ö. (2022). A Case of Juvenile Systemic Sclerosis and Congenital Pulmonary Airway Malformation Related Mucinous Adenocarcinoma of the Lung: Paraneoplastic Syndrome or Just a Coincidence?. Turk. J. Pediatr..

[30] Khimdas S., Harding S., Bonner A., Zummer B., Baron M., Pope J. (2011). Canadian Scleroderma Research Group Associations with Digital Ulcers in a Large Cohort of Systemic Sclerosis: Results from the Canadian Scleroderma Research Group Registry. Arthritis Care Res..

[31] Tiev K.P., Diot E., Clerson P., Dupuis-Siméon F., Hachulla E., Hatron P.-Y., Constans J., Cirstéa D., Farge-Bancel D., Carpentier P.H. (2009). Clinical Features of Scleroderma Patients with or without Prior or Current Ischemic Digital Ulcers: Post-Hoc Analysis of a Nationwide Multicenter Cohort (ItinérAIR-Sclérodermie). J. Rheumatol..

[32] Van Roon A.M., Huisman C.C., van Roon A.M., Zhang D., Stel A.J., Smit A.J., Bootsma H., Mulder D.J. (2019). Abnormal Nailfold Capillaroscopy Is Common in Patients with Connective Tissue Disease and Associated with Abnormal Pulmonary Function Tests. J. Rheumatol..

